# Novel prognostic impact and cell specific role of endocan in patients with coronary artery disease

**DOI:** 10.1007/s00392-024-02458-7

**Published:** 2024-05-13

**Authors:** Liang-Yu Lin, Ting-Ting Chang, Hsin-Bang Leu, Chin-Chou Huang, Tao-Cheng Wu, Ruey-Hsin Chou, Po-Hsun Huang, Wei-Hsian Yin, Wei-Kung Tseng, Yen-Wen Wu, Tsung-Hsien Lin, Hung-I Yeh, Kuan-Cheng Chang, Ji-Hung Wang, Chau-Chung Wu, Jaw-Wen Chen

**Affiliations:** 1 Division of Endocrinology and Metabolism, Department of Medicine, Taipei Veterans General Hospital, No. 201, Sec. 2, Shih-Pai Road, Taipei, 11217 Taiwan; 2 School of Medicine, National Yang Ming Chiao Tung University, Taipei, Taiwan; 3 Department and Institute of Pharmacology, National Yang Ming Chiao Tung University, Taipei, Taiwan; 4 Healthcare and Services Center, Taipei Veterans General Hospital, Taipei, Taiwan; 5 Division of Cardiology, Department of Medicine, Taipei Veterans General Hospital, Taipei, Taiwan; 6 Department of Critical Care Medicine, Taipei Veterans General Hospital, Taipei, Taiwan; 7 Cardiovascular Research Center, National Yang Ming Chiao Tung University, Taipei, Taiwan; 8 Division of Cardiology, Heart Center, Cheng-Hsin General Hospital, Taipei, Taiwan; 9 Department of Medical Imaging and Radiological Sciences, I-Shou University, Kaoshiung, Taiwan; 10 Division of Cardiology, Department of Internal Medicine, E-Da Hospital, Kaoshiung, Taiwan; 11 Division of Cardiology, Cardiovascular Medical Center and Department of Nuclear Medicine, Far-Eastern Memorial Hospital, New Taipei City, Taiwan; 12 Division of Cardiology, Department of Internal Medicine, Kaohsiung Medical University Hospital and Kaohsiung Medical University, Kaohsiung, Taiwan; 13 Mackay Memorial Hospital, Mackay Medical College, New Taipei City, Taiwan; 14 Division of Cardiology, Department of Internal Medicine, China Medical University Hospital, Taichung, Taiwan; 15 Graduate Institute of Clinical Medical Science, China Medical University, Taichung, Taiwan; 16 Department of Cardiology, Buddhist Tzu-Chi General Hospital, Tzu-Chi University, Hualien, Taiwan; 17 Division of Cardiology, Department of Internal Medicine, National Taiwan University College of Medicine and Hospital, Taipei, Taiwan; 18 Department of Primary Care Medicine, College of Medicine, National Taiwan University, Taipei, Taiwan; 19 Division of Cardiology, Department of Medicine, and Department of Medical Research, Taipei Medical University Hospital, 252 Wuxing St., Taipei, 11031 Taiwan Republic of China; 20 Cardiovascular Research Center, and School of Medicine, Taipei Medical University Hospital, Taipei Medical University, Taipei, Taiwan

**Keywords:** Cardiovascular disease, Coronary artery disease, Endocan, Endothelial progenitor cell

## Abstract

**Background:**

Both the clinical and mechanistic impacts of endocan were not well elucidated especially in coronary artery disease (CAD).

**Objective:**

This study aimed to investigate the prognostic and potential pathological role of endocan for cardiovascular (CV) events in stable CAD patients.

**Methods:**

A total of 1,071 stable CAD patients with previous percutaneous coronary intervention (PCI) were enrolled prospectively in a nationwide Biosignature study. Another cohort of 76 CAD patients with or without PCI were enrolled for validation. Baseline biomarkers including endocan level was measured and total CV events especially hard CV events (including CV mortality, non-fatal myocardial infection and stroke) during follow-up were identified. Circulating endothelial progenitor cells (EPCs) as an in vivo biological contributor to vascular repairment from CAD patients were used for the in vitro functional study.

**Results:**

After 24 months, there were 42 patients (3.92%) with hard CV events and 207 (19.3%) with total CV events in the study group. The incidence of both events was increased with the tertiles of baseline endocan level (hard events: 1.7%,3.4%, and 6.7% in 1st,2nd, and 3rd tertile respectively, p = 0.002; total events: 13.8%vs.16.2%vs.28.0%, p < 0.0001). Multivariate regression analysis revealed the independent association of endocan level with total and hard CV events. These findings were validated in another cohort with a 5-year follow-up. Furthermore, in vitro inhibition of endocan improved cell migration and tube formation capacities, and reduced cell adhesiveness of EPCs from CAD patients.

**Conclusions:**

Endocan might be a novel prognostic indicator, mechanistic mediator, and potential therapeutic target for clinical CAD.

**Graphical abstract:**

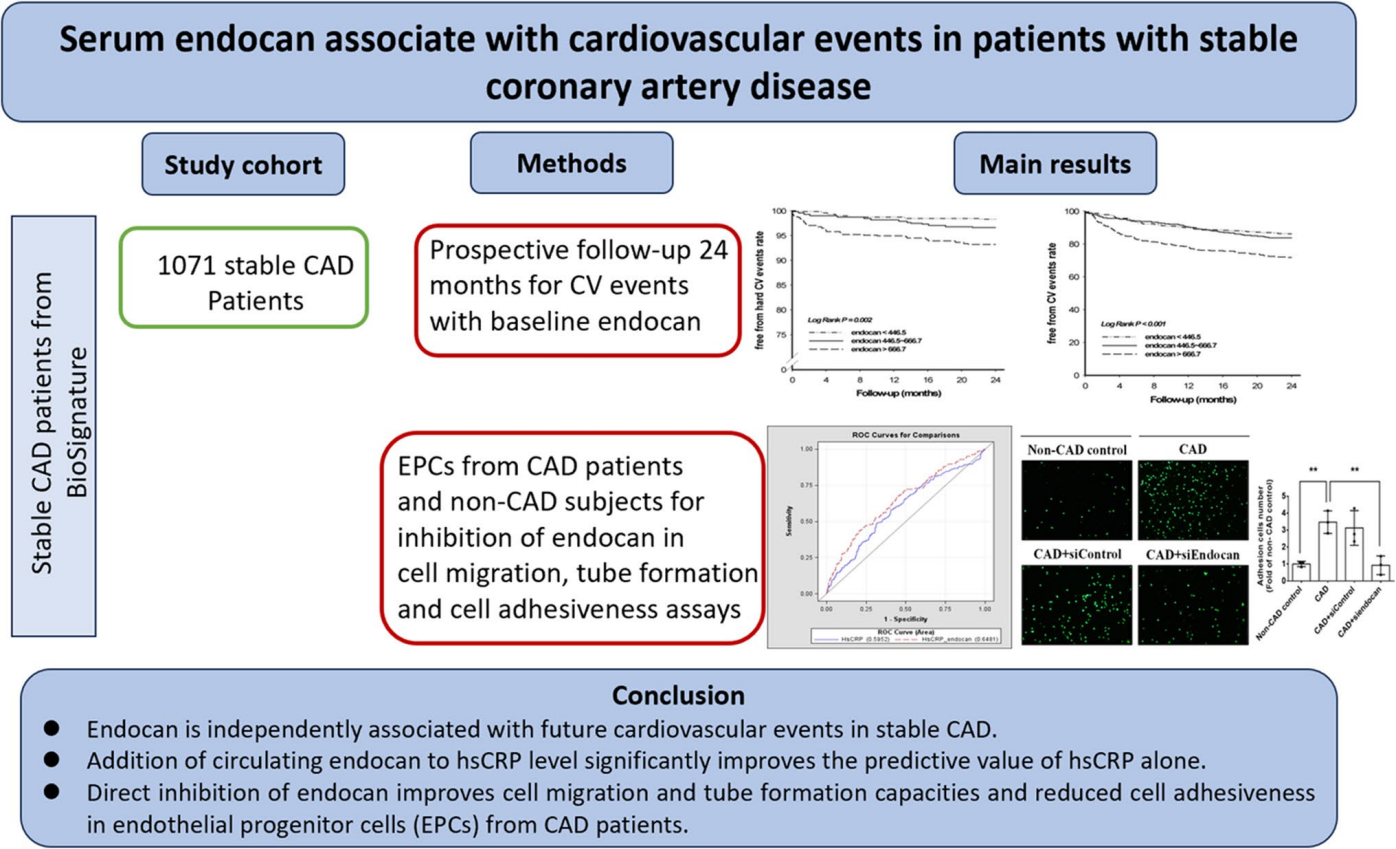

**Supplementary Information:**

The online version contains supplementary material available at 10.1007/s00392-024-02458-7.

## Introduction

Coronary artery disease (CAD) is one of the leading causes of death worldwide. Patients with stable CAD have an increased risk of future cardiovascular (CV) events. Given that the severity of coronary arterial stenosis cannot efficiently predict the risk of plaque rupture and subsequent CV events, proper risk stratification is critical to effective clinical management of these very-high risk patients [1, 2]. Although serial systemic biomarkers have been identified for CAD, their usefulness in stratifying future risk is limited [3–5]. Improved predictors for disease progression are required to determine appropriate medications and disease management strategies.

Inflammation is suggested to play a key role in the pathogenesis of atherosclerotic CV diseases [6–10]. There was a significant association between elevated inflammatory biomarkers, such as high-sensitive C-reactive protein (hsCRP), and the risk of atherothrombotic events in various disease settings [6–10]. In CAD patients with prior percutaneous coronary intervention (PCI), the incidence of all-cause death and hard CV events was still very high at 27.4% and 30.8%, respectively after 4.3 years follow-up [11]. While serum hsCRP may be a useful biomarker mainly for CAD patients with intermediate risk, new biomarkers are still required for clinical risk stratification in patients at very-high risk, such as CAD patients with PCI [12].

Increasing evidence suggests that individuals with CAD exhibit abnormalities in both the structure and function of their endothelial cells [13, 14]. Studies have highlighted the pivotal role of bone marrow-derived circulating endothelial progenitor cells (EPCs) in repairing endothelial damage and preserving endothelial function [15]. However, in CAD patients, the function of EPCs is compromised, leading to delays in vascular endothelial repair [16, 17]. Strategies aimed at restoring EPC function, including enhancing proliferation, tube formation, and migration in laboratory settings, hold promise for improving therapeutic outcomes [18].

Endocan (endothelial cell specific molecule-1) is a soluble dermatan sulfate proteoglycan comprising a 165 amino acid polypeptide; it may be secreted by vascular endothelial cells that are under the control of inflammatory cytokines. In addition to playing a significant role in the regulation of in vitro endothelial cell adhesion upon hypoxia, endocan was recognized as a novel endothelial marker for cancer and a new target for cancer therapy given its potential involvement in systemic inflammation, tumor-associated angiogenesis and cancer metastasis *in vi*vo. [19, 20] Besides, previous preliminary experimental data also found the upregulation of endothelial endocan expression in atherosclerotic plaques in vivo**.** [21] Given the cardinal role of endothelial dysfunction for atherogenesis and the endothelial cell specific nature of endocan, one may speculate the potential significance of endocan in the progression of clinical atherosclerosis for future CV events.

Indeed, endocan recently received increased attention as a potential marker of endothelial dysfunction for the presence of clinical atherosclerosis, and was suggested as a potential marker of CV events in patients with acute coronary syndrome [22–26] as well as in patients with chronic kidney disease [27, 28]. However, the mechanistic impacts of endocan to the development of CV events were yet established. A systematic evaluation of circulating endocan and its role in stable CAD was also lacking.

The current prospective study aimed to elucidate whether baseline circulating endocan levels could be associated with future CV outcomes at 24-months in stable CAD patients with prior PCI. Additional in vitro studies were also conducted to investigate the potential pathogenic role and mechanistic effects of endocan on EPCs retrieved from CAD patients.

## Methods

### Clinical study

#### Study population

The present Biosignature study was a nationwide prospective cohort study that was carried out in nine centers across Taiwan from October 2012 to May 2015 to identify risk factors among stable CAD patients at baseline. Detailed protocols for this study have been published previously [29]. Briefly, the inclusion criteria were patients with significant CAD, as documented by a coronary angiogram, a history of myocardial infarction, as evidenced by a 12-lead electrocardiography (ECG) or hospitalization, or a history of angina with ischemic ECG changes or a positive response to stress test. Patients who received at least one successful PCI with either coronary stenting or balloon angiography and were considered stable on medical treatment for at least one month, were enrolled in the study. Patients were excluded if (i) they had been hospitalized for unstable angina, acute coronary syndrome, acute myocardial infarction, acute cerebrovascular events or other acute CV events within the 3 months prior to enrollment, (ii) they planned to receive further coronary revascularization or interventional procedures for other CV diseases during the following one year period, (iii) they had significant malignancies or tumors requiring advanced medical or surgical therapy or both in the following one year, or (iv) they had other major systemic diseases requiring hospitalization or operation in the following one year period. In addition, patients with a life expectancy < 6 months (*e.g.* malignant metastatic neoplasm), and those receiving treatment with immunosuppressive agents were also excluded. The study was approved by the Health Authority, Independent Ethics Committee, and Independent Review Board (IRB) at each hospital, as well as the Joint IRB Ethics Committee Review Board in Taiwan. All participants provided informed consent prior to their inclusion within the study.

#### Validation cohort

An independent validation cohort, consisting of 76 stable CAD patients with or without PCI enrolled, was also investigated for comparison with the study patients. All patients had confirmed CAD, defined as stenosis of ≥ 50% in any major epicardial vessel on an angiography at the Taipei Veterans General Hospital (Taipei, Taiwan), between November 2011 and December 2015. The exclusion criteria were the same as for the study patients, and they were given contemporary medical treatment. They were prospectively followed up until September 2019 or until the occurrence of an adverse CV event, such as CV death, fatal or non-fatal myocardial infarction, ischemic stroke, or target vessel revascularization for recurrent angina.

#### Biochemical measurements

When patients first visited the out-patient clinic after their hospital discharge, blood samples were drawn after a minimum 8 h fast. Total cholesterol and triglyceride were measured by the enzymatic calorimetric method. Plasma high-density lipoprotein (HDL-c) and low-density lipoprotein (LDL-c) cholesterol were measured using the selective detergent method. Plasma creatinine was measured via standard methods and the estimated glomerular filtration rate (eGFR) was calculated using the MDRD formula. Plasma hsCRP, tumor necrosis factor-α (TNF-α), N-terminal of the prohormone brain natriuretic peptide (NT-proBNP) and endocan were detected using Milliplex MAP kit assays (Millipore Corporation, Darmstadt, Germany). In the validation cohort, plasma endocan was assessed using Sandwich ELISA kits (Abcam, Cambridge, MA, USA).

#### Clinical follow up for adverse events

All patients were regularly followed up and a trained study nurse performed data collection every 3 months for the first year and every 6 months from the second year onwards. The primary outcome was hard CV events, including CV mortality, non-fatal myocardial infarction and stroke. The secondary outcome was total CV events, including all-cause mortality, non-fatal myocardial infarction, stroke, hospitalization for coronary intervention, peripheral artery disease, heart failure and arrhythmia. Stroke was diagnosed if a focal deficit lasted for > 24 h. Maintenance therapy following PCI included aspirin (80–325 mg/day continuously) and clopidogrel (75 mg/day ≥ 6 months in patients with stents). Other medications were prescribed at the discretion of the attending physician.

### In vitrostudy

#### Human EPC isolation and culture

The blood sample was collected from the peripheral veins of an independent group of 3 patients with stable CAD and 3 non-CAD subjects. The detailed methodology for the culture of EPCs has been mentioned in our previous study [22, 30–32]. In brief, after blood was collected, the total mononuclear cells were separated by Histopaque-1077 (Sigma-Aldrich, 10,771, Darmstadt, Germany) and centrifuged at 500 × g at room temperature for 30 min. The mononuclear cells were cultured in endothelial cell basal medium (Lonza, CC-3156, Basel, Switzerland), with supplements including hydrocortisone, human fibroblast growth factors, vascular endothelial growth factor (VEGF), R3-insulin-like growth factor-1, ascorbic acid, human epidermal growth factor, gentamicin sulfate-amphotericin and 20% fetal bovine serum on fibronectin-coated 6-well plates. After culture for 4 ~ 5 days, the nonadherent cells were removed and the attached early EPCs appeared. At 2–4 weeks, some early EPCs continued to grow into colonies of late (out-growth) EPCs, usually with the monolayer cobblestone-like shape in a pattern typical of mature endothelial cells at confluence. Various immunofluroscence stainings may be used to characterize the late EPCs as indicated. Only the late EPCs under passage 3 were used for consequent EPC study.

#### Transfection of siRNA

EPCs were transfected with endocan siRNA (Santa Cruz Biotechnologies, sc-40543, Dallas, TX, USA) using Lipofectamine 2000 (Invitrogen, 12,252,011, Waltham, MA, USA) in culture medium.

#### Migration assay

The migratory function of EPCs was evaluated by a Boyden chamber assay (Transwell, Coster, San Diego, CA, USA). Briefly, the cells (1 × 10^4^ cells) were seeded on in the upper chambers of 24-well Transwell plates with a polycarbonate membrane (8-mm pores). Then, cells migrated toward the lower chamber containing 600 μL cultured medium with FBS at 37 °C in 5% CO_2_. After 18 h, migrated cells were fixed in 4% paraformaldehyde and stained with hematoxylin solution. Images were captured by a high-power (× 100) microscope.

#### Tube formation assay

The cells (1 × 10^4^ cells) were seeded into ECMatrix gel (Invitrogen, Carlsbad, CA, USA) in 96-well plates in 100 μL cultured medium with 10% FBS for 16 h at 37 °C in 5% CO_2_. Images were captured by high-power (× 40) microscope. The numbers of formed tubes of cells were calculated using Image-Pro Plus (Media Cybernetics, Inc. Rockville, MD, USA). The number of tube formation was evaluated by counting the total area of complete tubes formed by the cells in 6 representative fields.

#### Adhesion assay

THP-1 is a human monocytic cell line labeled with 10 Μm BCECF-AM (Invitrogen, B1170, Waltham, MA, USA) at 37 °C for 1 h in RPMI-1640 medium (Corning, Manassas, VA, USA). Confluent EPCs were incubated with THP-1 cells (5 × 10^5^ cells/mL) at 37 °C for 1 h. Nonadherent THP-1 cells were removed, and plates were gently washed with PBS. The number of adherent THP-1 cells was counted under a 200 × high-power field well using a fluorescent microscope (Zeiss, Axiovert 200 M; White Plains, NY, USA). Six randomly chosen high-power fields were counted per well.

#### Western blot analysis

Total cell or tissue lysates were extracted using lysis buffer, and proteins were separated in 8–12% (v/v) SDS-PAGE gels. After electrophoresis (Bio-Rad Laboratories, Hercules, CA, USA), the proteins were transferred onto PVDF membranes (Millipore, Darmstadt, Germany), and the membranes were incubated with anti-endocan (R&D Systems, AF1810, Minneapolis, MN, USA), VEGF (Santa Cruz Biotechnologies, sc-7269, Dallas, TX, USA), stromal cell-derived factor (SDF)-1 (Cell Signaling, Danvers, #3740, MA, USA), vascular cell adhesion molecule-1 (VCAM-1; Cell Signaling, #13,662, Danvers, MA, USA), intercellular adhesion molecule-1 (ICAM-1; Cell Signaling, #67,836, Danvers, MA, USA), E-selectin (Santa Cruz Biotechnologies, sc-137054, Dallas, TX, USA), anti-actin (Merck, 3,423,208, Darmstadt, Germany) at 4 ℃ overnight. After washing three times, the membranes were incubated with HRP-conjugated secondary antibodies for 1 h at room temperature. Finally, the membranes were visualized using the ECL kit.

#### Statistical analysis

Statistical analysis was performed using the Statistical Product and Service Solutions software, version 18.0. Descriptive statistics were expressed as the mean ± standard deviation for continuous variables, and as the number of cases and percentage (%) for categorical variables. Analyses of differences between groups were performed using the Pearson Chi Squared, Student’s *t*-test or one-way analysis of variance (ANOVA). The Cox proportional hazard model was used to assess the association between endocan and cardiovascular events, while adjusting for baseline cardiovascular risk factors and clinical variables. A two-tailed *P*-value of < 0.05 was considered statistically significant.

## Results

### Clinical study

#### Baseline clinical characteristics of study cohort

The baseline clinical characteristics of the 1,071 patients with stable CAD are summarized in Table 1. In our cohort, endocan level did not fit normal distribution (p < 0.001, examined by Kolmogorov–Smirnov). For further analysis, the patients were then divided into three groups (tertiles) according to their level of plasma endocan; 1st tertile (endocan < 466.5 pg/mL; n = 356 patients), 2nd tertile (endocan ≥ 466.5 to 666.7 pg/mL; n = 358 patients) and 3rd tertile (endocan > 666.7 pg/mL; n = 357 patients). The variables that differed significantly between endocan tertiles were: age, gender, body mass index, use of anti-coagulants, use of statins, eGFR and triglycerides. In addition, the baseline characteristics of the 1,071 control patients with stable CAD are summarized in Table 2, according to the presence of CV events. The variables that differed significantly between patients with or without total cardiovascular events were: current smoker, fasting glucose, white blood cell count, triglyceride and HDL-c.

**Table 1:** Baseline characteristics of study subjects according to the plasma endocan concentration tertile

Characteristics	All participants (n = 1071)	Plasma endocan (pg/mL)			p-value
		Tertile 1 (< 446.5) (n = 356)	Tertile 2 (446.5–666.7) (n = 358)	Tertile 3 (> 666.7) (n = 357)	
Age (years)	65.5 ± 12.0	61.1 ± 10.6	65.8 ± 11.7	69.4 ± 12.3	< 0.001
Male, no. (%)	913 (85.2)	316 (88.8)	307 (85.8)	290 (81.2)	0.017
Body mass index (kg/m^2^)	26.3 ± 4.4	26.8 ± 3.6	26.5 ± 3.8	25.6 ± 5.6	< 0.001
Hypertension, no. (%)	698 (65.2)	221 (62.1)	232 (64.8)	245 (68.6)	0.183
Diabetes mellitus, no. (%)	408 (38.1)	132 (37.1)	134 (37.4)	142 (39.8)	0.722
Current smoker, no. (%)	603 (56.3)	206 (57.9)	201 (56.2)	196 (54.9)	0.726
Use of anti-platelet, no. (%)	992 (92.6)	334 (93.8)	337 (94.1)	321 (89.9)	0.056
Use of anti-coagulant, no. (%)	28 (2.6)	3 (0.8)	11 (3.1)	14 (3.9)	0.029
Use of ACEi/ARB, no. (%)	667 (62.3)	228 (64.0)	227 (63.4%)	212 (59.4)	0.379
Use of statin, no. (%)	746 (69.7)	267 (75.0)	252 (70.4)	227 (63.6)	0.004
Fasting glucose (mg/dL)	121.3 ± 45.0	121.6 ± 41.7	123.9 ± 45.3	118.3 ± 42.5	0.247
eGFR (mL/min/1.73 m^2^)	74.5 ± 31.4	81.4 ± 25.6	77.6 ± 36.2	64.5 ± 29.0	< 0.001
WBC count (CUMM)	7389 ± 2317	7420 ± 2207	7406 ± 2418	7339 ± 2326	0.888
Total cholesterol (mg/dL)	163.1 ± 35.7	164.3 ± 34.9	164.4 ± 36.6	160.5 ± 35.5	0.256
Triglyceride (mg/dL)	134.5 ± 83.7	145.6 ± 90.2	139.5 ± 87.1	118.4 ± 70.2	< 0.001
LDL-c (mg/dL)	94.1 ± 28.8	94.7 ± 28.3	94.7 ± 29.7	93.0 ± 28.4	0.667
HDL-c (mg/dL)	41.9 ± 10.7	41.3 ± 10.2	41.5 ± 10.5	43.0 ± 11.3	0.078
hsCRP (mg/dL)	0.371 ± 0.955	0.343 ± 0.948	0.301 ± 0.531	0.458 ± 1.211	0.147
NT-pro-BNP (pg/mL)	392.4 ± 854.9	218.4 ± 292.4	309.3 ± 415.6	649.3 ± 1354.5	< 0.001
TNF-α (pg/mL)	4.56 ± 5.27	3.30 ± 3.37	5.05 ± 6.45	5.37 ± 5.45	< 0.001
Endocan (pg/mL)	656.2 ± 500.6	338.5 ± 78.3	547.7 ± 60.2	1081.7 ± 670.2	< 0.001

*ACEi/ARB* Angiotensin converting enzyme inhibitor/Angiotensin receptor blocker, *eGFR* estimated glomerular filtration rate, *HDL-c* high-density lipoprotein cholesterol, *hsCRP* high sensitive C-reactive protein, *LDL-c* low-density lipoprotein cholesterol, *NT-pro-BNP* N-terminal of the prohormone brain natriuretic peptide, *TNF-α* tumor necrosis factor-α, *WBC* white blood cells

**Table 2:** Baseline characteristics of 1,071 stable coronary artery disease patients with and without cardiovascular events

Variables	Cardiovascular events		p-value
	No (n = 864)	Yes (n = 207)	
Age (years)	65.8 ± 11.8	64.0 ± 13.1	0.065
Male, no. (%)	739 (85.5)	174 (84.1)	0.591
Body mass index (kg/m^2^)	26.3 ± 4.6	26.4 ± 3.9	0.727
Hypertension, no. (%)	566 (65.5)	132 (63.8)	0.637
Diabetes mellitus, no. (%)	321 (37.2)	87 (42.0)	0.194
Current smoker, no. (%)	468 (54.2)	135 (65.2)	0.004
Use of anti-platelet, no. (%)	797 (92.3)	195 (94.2)	0.333
Use of anti-coagulant, no. (%)	23 (2.7)	5 (2.4)	0.842
Use of ACEi/ARB, no. (%)	543 (62.9)	124 (59.9)	0.433
Use of statin, no. (%)	602 (69.7)	144 (69.6)	0.975
Fasting glucose (mg/dL)	119.0 ± 41.5	130.7 ± 56.6	0.005
eGFR (mL/min/1.73 m^2^)	75.0 ± 28.7	72.4 ± 40.7	0.384
WBC count (CUMM)	7308 ± 2264	7707 ± 2495	0.039
Total cholesterol (mg/dL)	162.5 ± 34.7	165.5 ± 39.5	0.317
Triglyceride (mg/dL)	131.2 ± 78.8	148.5 ± 100.6	0.022
LDL-C (mg/dL)	93.4 ± 28.8	97.1 ± 28.6	0.097
HDL-C (mg/dL)	42.4 ± 10.8	40.0 ± 9.9	0.003
hsCRP (mg/dL)	0.317 ± 0.769	0.570 ± 1.430	0.029
NT-pro-BNP (pg/mL)	347.9 ± 858.5	578.1 ± 815.7	< 0.001
TNF-α (pg/mL)	4.31 ± 5.17	5.47 ± 5.54	0.016
Endocan (pg/mL)	619.6 ± 424.2	808.9 ± 720.3	< 0.001

*ACEi/ARB* Angiotensin converting enzyme inhibitor/Angiotensin receptor blocker, *eGFR* estimated glomerular filtration rate, *HDL-c* high-density lipoprotein cholesterol, *hsCRP* high sensitive C-reactive protein, *LDL-c* low-density lipoprotein cholesterol, *NT-pro-BNP* N-terminal of the prohormone brain natriuretic peptide, *TNF-α* tumor necrosis factor-α, *WBC* white blood cells

#### Clinical outcomes

**Fig. 1:**
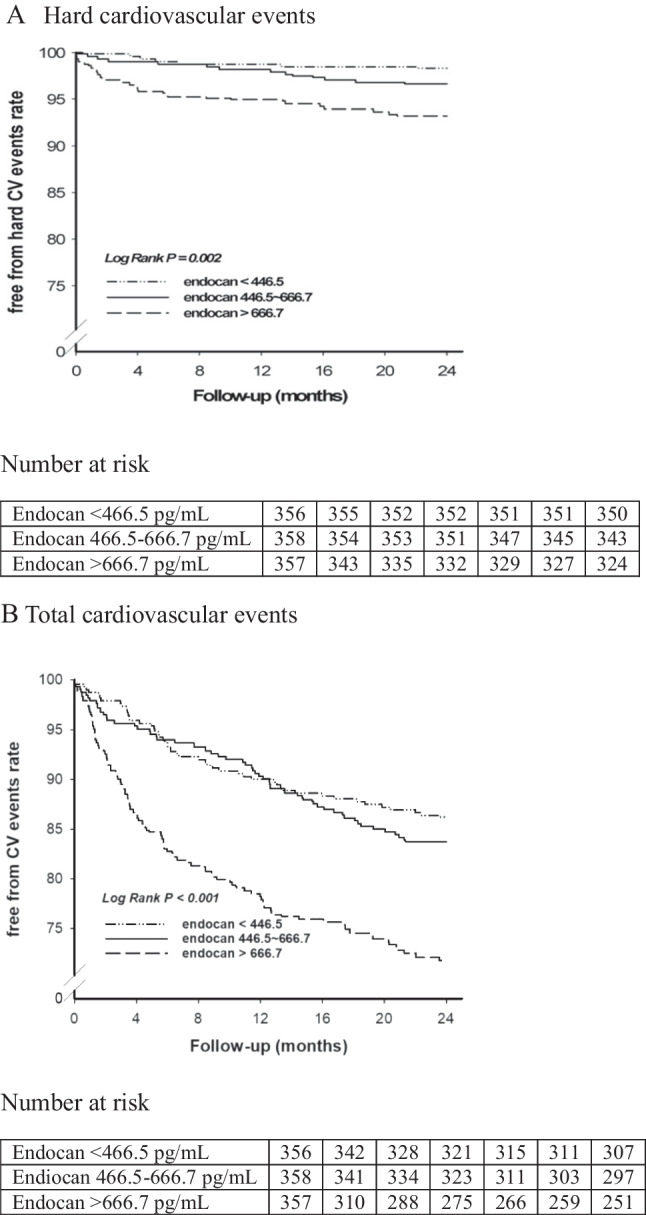
Endocan and prognosis in patients with stable coronary artery disease. Kaplan–Meier curve displaying the (**A**) hard and (**B**) total cardiovascular event-free rates according to the tertile of endocan levels

All enrolled patients were followed up until the first onset of CV events or for 24 months, whichever came first. During follow-up there were a total of 207 (19.3%) CV events and 42 (20.3%) hard CV events, including 24 (2.2%) non-fatal myocardial infarctions, 6 (0.6%) non-fatal strokes and 12 (1.2%) deaths due to CV causes. For the primary outcome (hard CV events), there were 6 events in tertile 1, 12 in tertile 2 and 24 in tertile 3 (p = 0.002, ANOVA; Table 3). The Kaplan–Meier analysis showed that the incidence of hard CV events was 1.7% in tertile 1, 3.4% in tertile 2 and 6.7% in tertile 3 (log-rank test p = 0.002; Fig. 1A). For the secondary outcome (total CV events), there were 49 events in tertile 1, 58 in tertile 2 and 100 in tertile 3 (p < 0.0001, ANOVA; Table 3) The Kaplan–Meier analysis showed that the incidence of total CV events was 13.8% in tertile 1, 16.2% in tertile 2, and 28.0% in tertile 3 (log-rank test p < 0.0001; Fig. 1B). Subgroup analysis revealed similar CV event occurrence for patients with type 2 diabetes and stable CAD (Online Fig. 1).

**Table 3:** 24-month cardiovascular outcomes according to endocan tertiles

Outcomes, no. (%)	Plasma endocan (pg/mL)			p-value
	Tertile 1 (< 446.5) (n = 356)	Tertile 2 (446.5–666.7) (n = 358)	Tertile 3 (> 666.7) (n = 357)	
Total CV events	49 (13.8)	58 (16.2)	100 (28.0)	< 0.001
Hard CV events	6 (1.7)	12 (3.4)	24 (6.7)	0.002
CV mortality	0 (0.0)	1 (0.3)	11 (3.1)	< 0.0001
Non-fatal MI	5 (1.4)	9 (2.5)	10 (2.8)	0.413
Non-fatal stroke	1 (0.3)	2 (0.6)	3 (0.8)	0.606
Unstable angina	41 (11.5)	42 (11.7)	49 (13.7)	0.613
Heart failure	2 (0.6)	2 (0.6)	15 (4.2)	< 0.001
PAD	0 (0.0)	2 (0.6)	12 (3.4)	< 0.001

*CV* cardiovascular, *MI* myocardial infarction, *PAD* peripheral artery disease

#### Plasma endocan was an independent predictor for CV events

The association between the increase in endocan (in tertiles) and age, current smoking status, fasting glucose level, white blood cell counts, triglyceride, HDL-c, hsCRP, NT-proBNP, TNF-α and the risk of CV events was subsequently investigated (Table 4). Table 4A shows that the increase in endocan (hazard ratio [HR], 2.049; 95% confidence interval [CI], 1.637–4.229, p = 0.001), fasting glucose (HR, 1.009; 95% CI, 1.005–1.014, p < 0.001), and NT-proBNP (HR, 1.000; 95% CI, 1.000–1.000, p = 0.006) were significant predictors of hard CV events in the univariate analysis. Only the increase in endocan (HR, 1.774; 95% CI, 1.118–2.813, p = 0.015) and fasting glucose level (HR, 1.011; 95% CI, 1.005–1.017, p < 0.001) were independent predictors for hard CV events after adjusting for confounding factors in the multivariate analysis.

**Table 4:** Cox hazard regression analysis to predict (A) hard and (B) total cardiovascular events

Variables	Univariate		Multivariate*	
	HR [95% CI]	P value	HR [95% CI]	p-value
A. Hard cardiovascular events				
Endocan (pg/mL)	1.001 [1.000–1.001]	< 0.001	1.000 [1.000–1.001]	0.062
Endocan tertile	2.049 [1.351–3.1074]	< 0.001	1.774 [1.118–2.813]	0.015
Age (years)	1.004 [0.979–1.030]	0.749		
Current smoking	1.257 [0.674–2.344]	0.471		
Fasting glucose (mg/dL)	1.009 [1.005–1.014]	< 0.001	1.011 [1.005–1.017]	< 0.001
WBC(CUMM)	1.000 [1.000–1.000]	0.085	1.000 [1.000–1.000]	0.609
Triglyceride (mg/dL)	1.000 [0.996–1.004]	0.996		
HDL-c (mg/dL)	0.987 [0.958–1.018]	0.407		
hsCRP (mg/dL)	1.157 [0.982–1.364]	0.082	1.072 [0.872–1.317]	0.509
NT-proBNP (pg/mL)	1.000 [1.000–1.000]	0.006	1.000 [1.000–1.000]	0.113
TNF-α (pg/mL)	1.023 [0.975–1.073]	0.349		
B. Total cardiovascular events				
Endocan (pg/mL)	1.000 [1.000–1.001]	< 0.001	1.000 [1.000–1.001]	< 0.001
Endocan tertile	1.555 [1.306–1.851]	< 0.001	1.797 [1.452–2.225]	< 0.001
Age (years)	0.988 [0.977–0.999]	0.040	0.978 [0.964–0.992]	0.003
Current smoking	1.514 [1.137–2.015]	0.005	1.438 [1.022–2.024]	0.037
Fasting glucose (mg/dL)	1.005 [1.002–1.007]	< 0.001	1.003 [1.000–1.006]	0.045
WBC(CUMM)	1.000 [1.000–1.000]	0.015	1.000 [1.000–1.000]	0.468
Triglyceride (mg/dL)	1.002 [1.001–1.003]	0.007	1.001 [0.999–1.003]	0.158
HDL-c (mg/dL)	0.980 [0.966–0.994]	0.004	0.992 [0.976–1.009]	0.365
hsCRP (mg/dL)	1.170 [1.070–1.280]	< 0.001	1.083 [0.958–1.224]	0.204
NT-proBNP (pg/mL)	1.000 [1.000–1.000]	0.001	1.000 [1.000–1.000]	0.187
TNF-α (pg/mL)	1.029 [1.007–1.052]	0.010	1.013 [0.989–1.038]	0.289

*CI* confidence interval, *HDL-c* high-density lipoprotein cholesterol, *HR* hazard ratio, *hsCRP* high sensitive C-reactive protein, *LDL-c* low-density lipoprotein cholesterol, *NT-pro-BNP* N-terminal of the prohormone brain natriuretic peptide, *TNF-α* tumor necrosis factor-α, *WBC* white blood cells

^*^adjusted p < 0.1

**Fig. 2:**
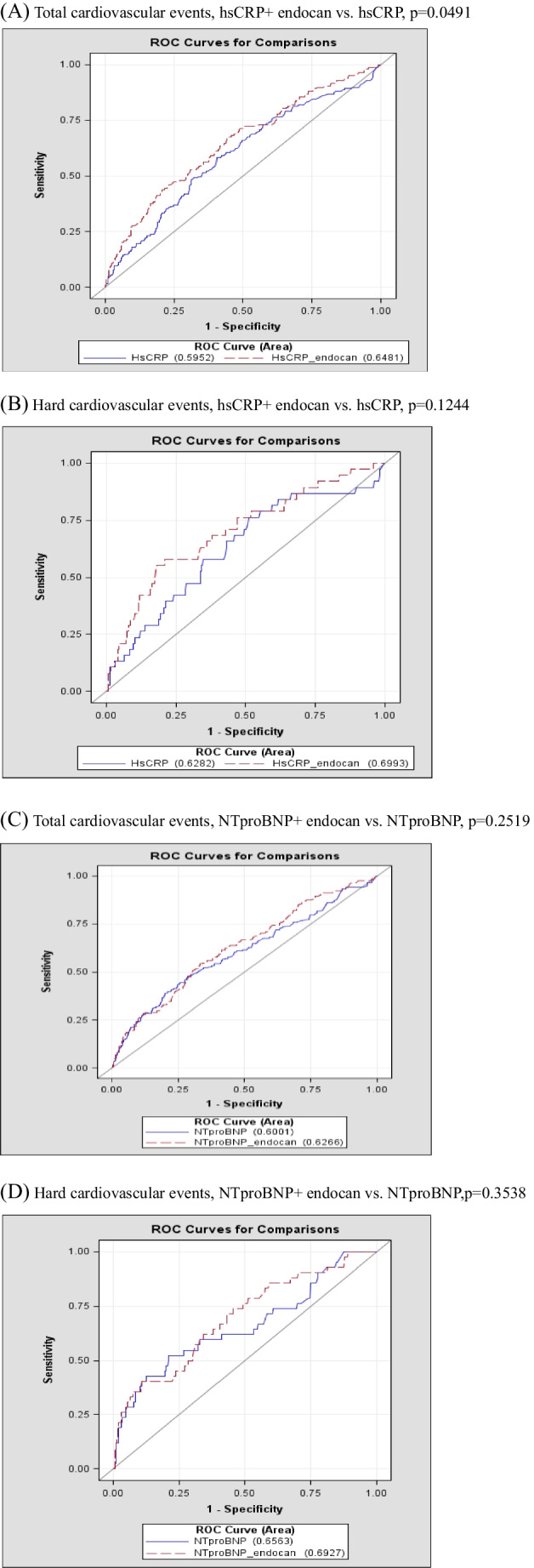
**A** Receiver operating characteristic (ROC) curve analysis using baseline endocan and high sensitivity C-reactive protein (hsCRP) for predicting the total cardiovascular events. The area under the curve (AUC) of endocan and hsCRP were 0.6481 and 0.5952, respectively. (**B**) ROC curve analysis using baseline endocan and hsCRP for predicting hard cardiovascular events. The AUC of endocan and hsCRP were 0.6993 and 0.6282, respectively. (**C**) ROC curve analysis using baseline endocan and N-terminal of the prohormone brain natriuretic peptide (NT-proBNP) for predicting total cardiovascular events. The AUC of endocan and NT-proBNP were 0.6001 and 0.6266, respectively. (**D**) ROC curve analysis using baseline endocan and NT-proBNP for predicting hard cardiovascular events. The AUC of endocan and NT-proBNP were 0.6563 and 0.6927, respectively

Table 4B shows that the increase in endocan (HR, 1.555; 95% CI, 1.306–1.851, p < 0.001), age (HR, 0.988; 95% CI, 0.977–0.999, p = 0.004), current smoking status (HR, 1.514; 95% CI, 1.137–2.015, p = 0.005), fasting glucose (HR, 1.005; 95% CI, 1.002–1.007, p < 0.001), white blood cell count (HR, 1.000; 95% CI, 1.000–1.000, p = 0.015), triglyceride (HR, 1.002; 95% CI, 1.001–1.003, p = 0.007), HDL-c (HR, 0.980; 95% CI, 0.966–0.994, p = 0.004), hsCRP (HR, 1.170; 95% CI, 1.070–1.280, p < 0.001), NT-proBNP (HR, 1.000; 95% CI, 1.000–1.000, p = 0.001) and TNF-α.(HR, 1.029; 95% CI, 1.007–1.052, p = 0.010) were independent predictors for total CV events in the univariate analysis. However, the increase in endocan (HR, 1.797; 95% CI, 1.452–2.225, p < 0.001), age (HR, 0.978; 95% CI, 0.964–0.992, p = 0.003), current smoking status (HR, 1.438; 95% CI, 1.022–2.024, p = 0.037) and fasting glucose level (HR, 1.003; 95% CI, 1.000–1.006, p = 0.045) were the only independent predictors for total CV events after adjusting for confounding factors in the multivariate analysis. Furthermore, receiver operating characteristic (ROC) curve analysis was performed to predict the total and hard CV events. The predictive value (area under the curve) of endocan was 0.6193 and 6514 for total and hard CV events respectively. The combination of endocan with hsCRP significantly improved the predictive value of hsCRP alone (area under the curve 0.684 vs. 0.5952; *p* = 0.0491) for total CV events (Fig. 2A) but not for hard CV events (area under the curve 0.6993 vs. 0.6282; *p* = 0.1244) (Fig. 2B). This add-on effect of endocan did not find in the combination of endocan and NT-proBNP by ROC curve analysis (Fig. 2C and D).

#### Subgroup analysis for the risk of CV events

**Fig. 3:**
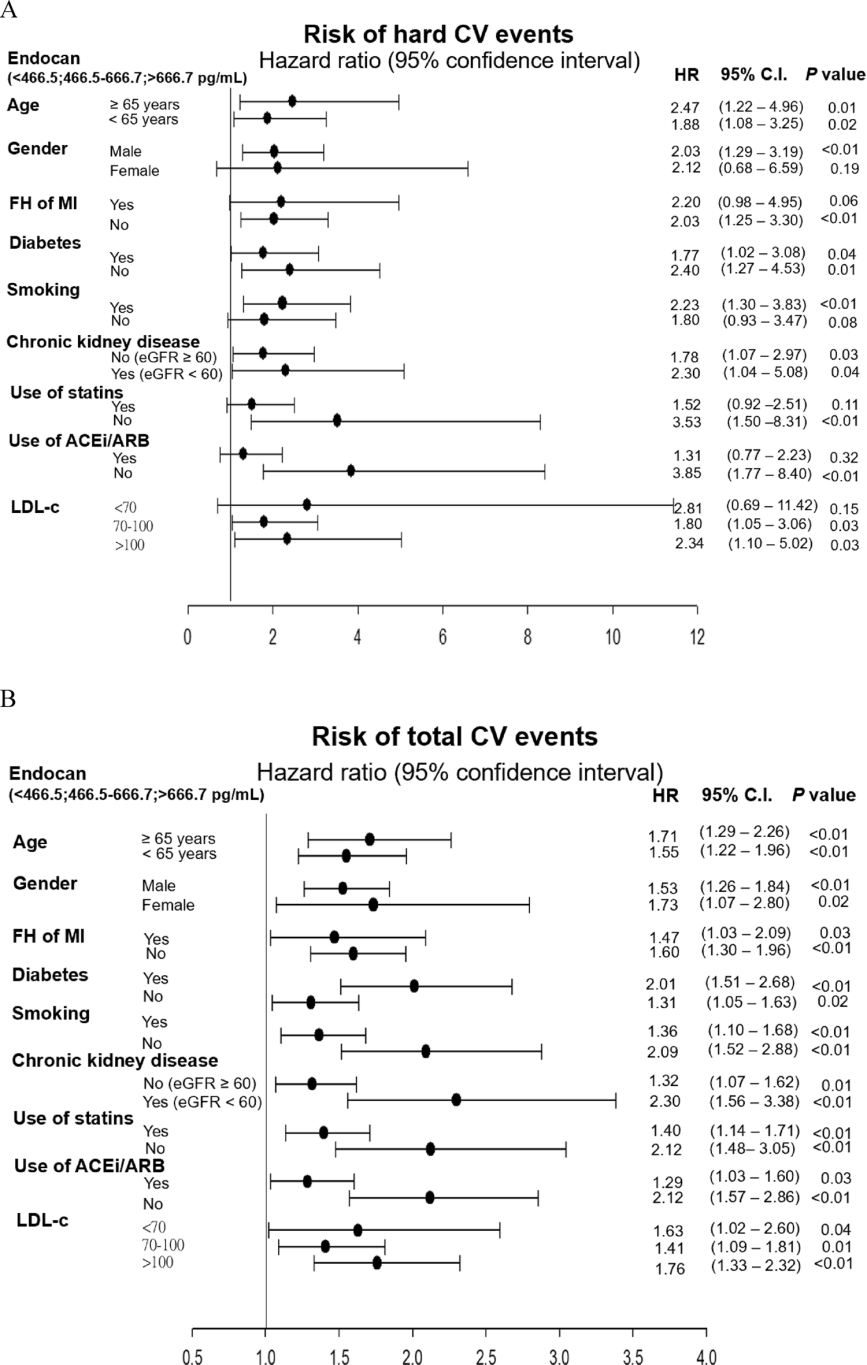
Hazard ratios (HRs) of endocan levels and the risk of (**A**) hard and (**B**) total cardiovascular events following subgroup analysis. CI, confidence interval

The subgroup analyses were preset for age, gender, family history of myocardial infarction, diabetes, smoking, chronic kidney disease, use of statins and ACEi/ARB, and LDL-c (Fig. 3). For each patient category, the relative risk (HR) of hard CV events (Fig. 3A) or total CV events (Fig. 3B) were calculated for tertile 3 patients and compared with tertile 1 patients. The risk of hard CV events was significantly increased in patients with higher serum endocan levels irrespective of their age, the presence of diabetes or the presence of chronic kidney disease. However, higher plasma endocan levels were associated with a significantly increased risk of hard CV events, primarily in male patients, those without a family history of myocardial infarction, smokers, those not using statins or ACEi/ARB, and in patients with a baseline serum LDL-c level ≥ 70 mg/dL (Fig. 3A). The risk of total CV events was universally increased in patients with higher plasma endocan levels irrespective of their age, gender, family history of myocardial infarction, diabetes, smoking status, chronic kidney disease, the use of statins and ACEi/ARB, or serum LDL-c level (Fig. 3B).

#### Validation study for endocan in patients with stable CAD

**Fig. 4:**
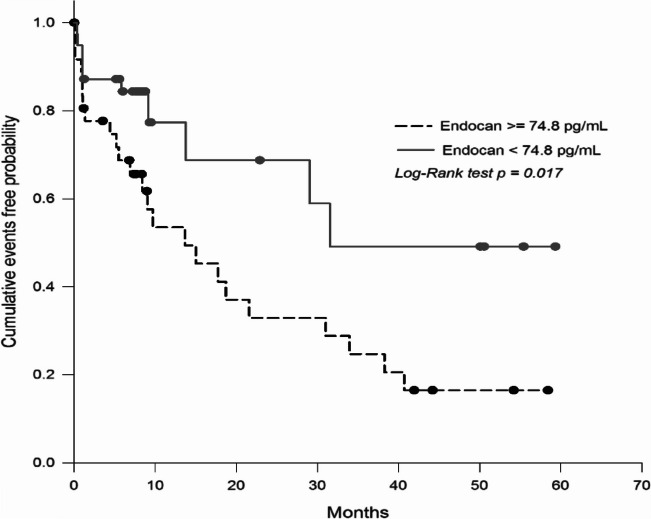
Endocan and prognosis in the validation cohort patients with stable coronary artery disease. Kaplan–Meier curve displaying the major adverse cardiovascular event (MACE)-free rates according to the medium endocan level

In the validation cohort of stable CAD patients, plasma endocan levels were measured by ELISA (quality controls were performed). A total of 76 patients, 53 men (69.7%), aged 65.7 ± 10.7 years, were available for further analysis. The demographic and biochemical characteristics of the validation cohort are presented in Online Table 1. Given the limited number of the patients, to ensure the adequate cohort for analysis, the validation cohort subjects were divided into two groups according to their medium plasma endocan level (74.8 pg/mL) at baseline. Patients with higher endocan levels were significantly older (p < 0.001) and had an increased history of hypertension (p = 0.002), increased fasting glucose levels (p = 0.048), and reduced eGFR (p = 0.016). During the average 5-year follow-up period, 35 (46.1%) CAD patients experienced adverse events. As shown in Fig. 4, there were 24 events in the endocan ≥ 74.8 pg/mL group and 11 events in the endocan < 74.8 pg/mL group (p = 0.003) (Online Table 1). Kaplan–Meier analysis revealed the incidence of CV events was 63.1% and 28.9%, respectively (log-rank test p = 0.017) (Fig. 4).

### In vitrostudy

#### Endocan inhibition reverses the impaired cell function in EPCs from CAD patients

**Fig. 5:**
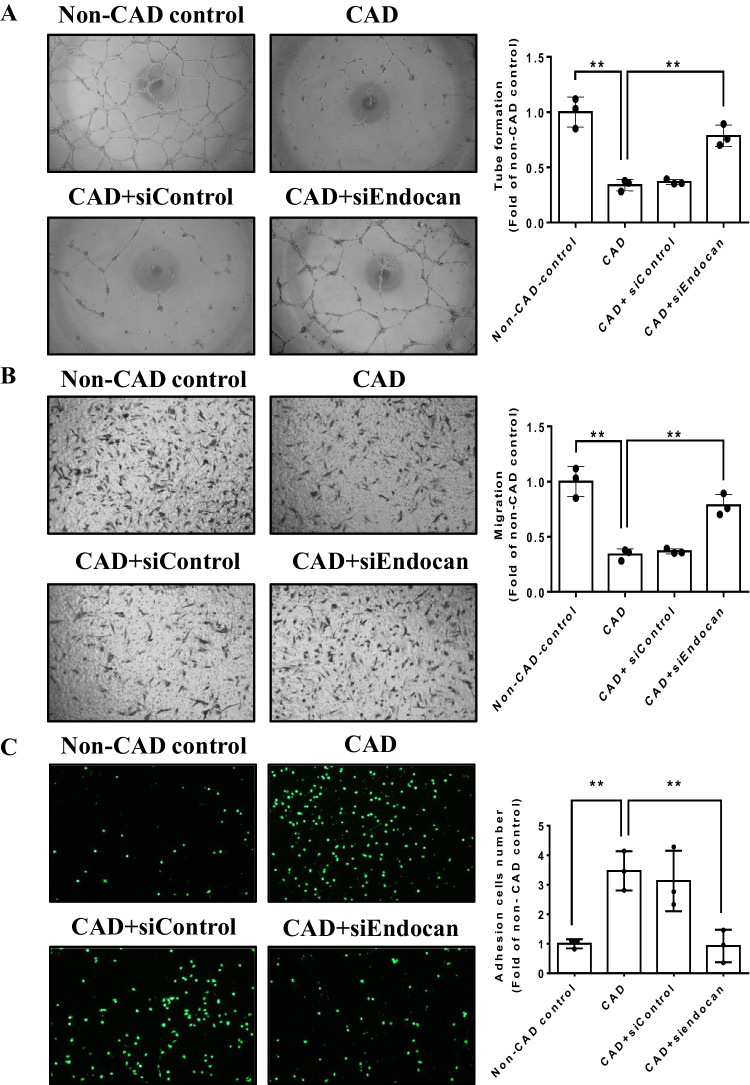

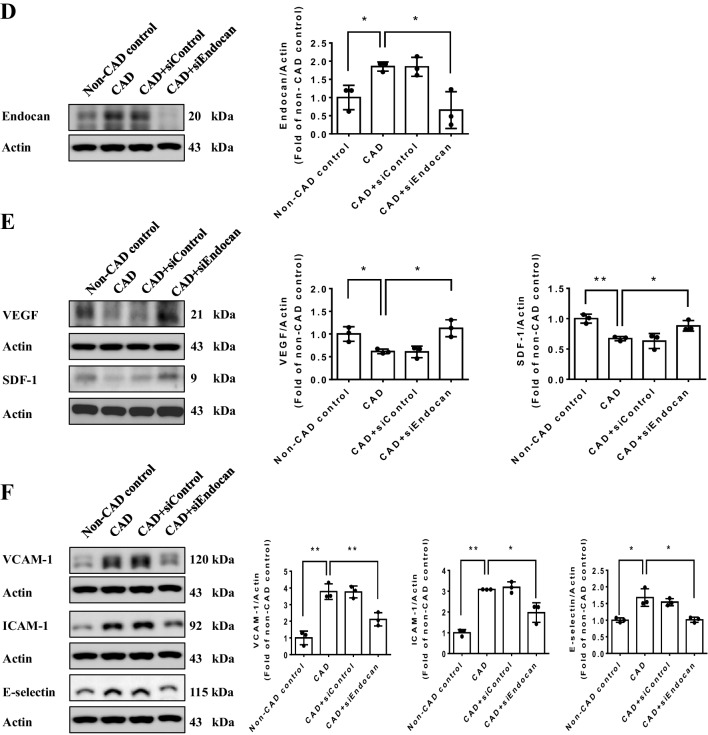
Inhibition of endocan by siRNA improved cell function and reduced adhesiveness of endothelial progenitor cells (EPCs) from CAD patients. (**A** and **B**) Inhibition of endocan improved tube formation and migration abilities in EPCs (n = 3). (**C**) Inhibition of endocan reduced adhesiveness of EPCs (n = 3). (**D** to **F**) Western blotting and statistical analysis of endocan, VEGF, SDF-1, VCAM-1, ICAM-1 and E-selectin expression in EPCs (n = 3). CAD, Coronary artery disease; EPCs, Endothelial progenitor cells, ICAM-1, Intercellular adhesion molecule-1; SDF-1, Stromal cell-derived factor-1; VCAM-1, Vascular cell adhesion molecule-1; VEGF, Vascular endothelial growth factor. * p < 0.05, ** p < 0.01

The tube formation and migration abilities were impaired in EPCs from CAD patients compared to the EPCs from non-CAD control subjects. The inhibition of endogenous endocan by siRNA significantly improved cell functions in EPCs from CAD patients (Fig. 5A and B). In EPCs from CAD patients, the adhesiveness of cells to THP-1 monocytic cells was attenuated by the administration of endocan siRNA (Fig. 5C). The EPCs from CAD patients had increased endocan expression and the expression of endogenous endocan was markedly reduced by endocan siRNA (Fig. 5D). In addition, the expressions of angiogenic proteins, such as VEGF and SDF-1 were increased in the endocan knockdown group in EPCs from CAD patients (Fig. 5E). On the other hand, adhesion molecules, such as VCAM-1, ICAM-1, and E-selectin were increased in EPCs from CAD patients and were decreased in the endocan siRNA-treated group (Fig. 5F). Taken together, these in vitro findings suggested that the inhibition of endogenous endocan may reverse the damaged cell function by up-regulating angiogenic proteins and down-regulating adhesion molecules in EPCs from CAD patients.

## Discussion

The primary findings of the present study indicate an association between baseline endocan levels and clinical CV outcomes in patients with stable CAD. To the best of our knowledge, this is the first large prospective study to show the prognostic impacts of circulating endocan in stable CAD patients. The prognostic significance of endocan could be also validated by different analyses in another independent patient cohort with an even longer follow-up period. Interestingly, compared to that in patients without events, baseline lipid profiles such as serum triglyceride was increased and HDL-c level was reduced, and baseline inflammatory profiles including hsCRP, NT-pro-BNP, and TNF-α were increased in patients with future events. However, only baseline endocan levels and baseline fasting glucose levels could independently predict total and hard CV events by multivariate Cox hazard regression analysis. On the other hand, circulating EPCs are the endothelial-like cells for vascular repairment and angiogenesis, which were used to investigate the potential pathological impact of endogenous endocan on vascular cells and function in vitro. Compared to that from non-CAD subjects, EPCs from CAD patients had increased endocan expression with impaired function. The inhibition of endogenous endocan reversed the dysfunction of EPCs with up-regulating angiogenic proteins, down-regulating inflammatory adhesion molecules, and attenuating the cell adhesiveness as an in vitro sign of atherogenesis. Taken together, our findings suggest the clinical significance and the potentially pathologic role of endocan in stable CAD. Future endocan-focused risk evaluation and therapeutic strategies may be considered in CAD patients at very-high risk of future events.

### Clinical significance of endocan in different diseases

It has been suggested that endocan may regulate major processes, such as cell adhesion, tumor progression, inflammation disorders and angiogenesis both in vitro or in vivo [33–35]. It has wide biological activity in different clinical settings and is considered a potential biomarker in cancer and sepsis [36–39]. It was also indicated that circulating endocan could be related to the severity of CAD [30, 31], to the presence of CAD in hypertensive patients [22, 30–32], and to the presence of diabetes mellitus [40–42]. The current findings further indicated the independent prognostic impact of circulating endocan on future CV events in stable CAD patients with prior PCI, which may be in line with the previous findings about the association of endocan with clinical outcomes in chronic kidney disease patients [27, 28], and in patients with acute coronary syndrome [23–26, 43]. Taken together, endocan might be a potential marker for patients at very high CV risk.

However, different from the findings of the above-mentioned studies including ours, there was no correlation between serum endocan and systemic inflammatory atherosclerosis markers such as hsCRP, adiponectin, and carotid intimal thickness in obesity [44]. Endocan seems not the universal marker of atherosclerosis especially in the context of obesity. Other endothelial as well as inflammatory markers may be considered in individual disease cohort. While circulating endocan might be secreted from activated vascular endothelial cells upon various stimulations, its significance to endothelial as well as systemic inflammation should be adjusted individually for different clinical diseases [45, 46].

Besides, the multiple and various medications used for different diseases might modify circulating inflammatory biomarkers such as hsCRP and others. Our recent unpublished data showed that the circulating endocan level may be also modified by medications. We had measured circulating endocan in a small number cohort (n = 180) in 2012 (unpublished data) to investigate the association of blood glucose status and levels of endocan. We found that a significant decrease the levels of endocan in patients with diabetes under oral hypoglycemic agent control (1.225 ± 0.624 ng/ml) compared with healthy subjects (1.765 ± 0.741 ng/ml). It is then possible that the multiple medications used may variously modify and further reduce the circulating endocan level in the current study patients with CAD. Further clinical study is indicated to investigate the individual medication that may modify endocan level before the standard endocan level could be universally applied in different clinical diseases.

### Clinical significance of endocan versus hsCRP in CAD

Inflammation plays an importance role in pathogenesis of stable CAD. Cytokines of hs-CRP and TNF-alpha represent different degrees of inflammation in human body. In addition, NT-proBNP is a marker of cardiac strength for heart failure and CAD is the most important cause of congestive heart failure. Therefore, we initiated the study to assess the predictive power of a novel biomarkers – endocan compared with these well-adapted biomarkers for future CV events in stable CAD patients. Among them, hsCRP is the most popular systemic inflammatory marker for stable CAD patients including Asian patients [47, 48]. However, while mainly produced in the liver following inflammatory insults, hsCRP is usually a surrogate biomarker for CV disease. Many contemporary CV medications, such as statins, ACEi/ARB and others have been shown to modify serum hsCRP levels [49], and consequently alter its prognostic impact in CAD patients under medical treatment. Given the inconsistency of clinical data from CAD patients, it is currently suggested that hsCRP may be used for primary prevention and risk stratification mainly in the intermediate risk cohorts, such as the subjects in the JUPITER trial [49]. The usefulness of hsCRP for the prediction of CV outcomes in patients with stable CAD remains controversial. Observational studies have suggested that periprocedural elevated hsCRP levels could be an independent risk factor for outcomes after PCI. However, it was also reported that there was no association between hsCRP levels and the need for repeat revascularization in PCI patients treated with drug-eluting stents [50]. In the present study, even though it was increased at baseline in CAD patients with subsequent clinical events, circulating hsCRP was not an independent risk factor for CV outcomes. In line with previous studies, the findings of the current study support the call for more vascular-related biomarkers, such as endocan, for contemporary risk stratification in stable CAD patients.

It has been reported that endocan levels could be strongly correlated with hsCRP in subjects with untreated essential hypertension [22]. Conversely though, Qui et al*.* did not find that endocan correlated with hsCRP in subjects with acute myocardial infarction [25]. In the present study, although both markers were significantly increased in at risk CAD patients, baseline endocan levels were not significantly associated with hsCRP. Endocan, not hsCRP, was a significant independent biomarker for future CV events. In addition, the combination of baseline endocan and hsCRP levels did provide further risk stratification compared with hsCRP alone, for total CV events in stable CAD patients. Therefore, while mainly derived from vascular endothelial cells, endocan as opposed to hsCRP and other systemic inflammatory biomarkers such as TNF-α, may be more specific for clinical outcomes in CAD patients.

### Prognostic impacts of endocan on total versus. hard CV events

Interestingly, in the current study, while endocan was a significant predictor of both total and hard events, it seemed more consistent for total rather than for hard CV events. There were no differences in the subgroup analyses for total CV events. However, in the sub-group analyses for hard CV events, the prognostic impact of endocan was mainly in patients who were male, had relatively higher baseline LDL-c levels, and were without statins or ACEi/ARB treatment, suggesting its particular effects in patients at relative high risk without adequate medical control. One possibility is that medications with potent vascular protection, such as statins and ACEi/ARB (used in 69.7% and in 62.3% of the study patients, respectively), may modify vascular inflammation and alter the prognostic impact of endocan, especially for vascular associated hard events. In fact, the use of statins was much less frequent in patients with highest endocan level in the 3rd tertile (63.6%) than in patients in the 2nd (70.4%) and in the 1st tertile (75.0%, p = 0.004), suggesting the potential impacts of statin use on baseline endocan levels and related hard CV events. Further investigation is required to clarify these issues.

### Potential pathological role of endocan in CAD

Though the implications of circulating endocan have been much evaluated in different clinical diseases, the pathological role of endocan in vascular inflammation should be further investigated. It was suggested that endocan may be a “vasculo-protective” molecule under normal physiological conditions. Intrinsic endocan may modulate endothelial cell behavior and promote vascular outgrowth by enhancing VEGF bioavailability in vivo [22]. On the other hand, exogenous administration of endocan could protect the endothelium from the migration and proliferation of inflammatory cells in vitro [17]. Once the endothelium is inflamed, some cytokines may activate endothelial endocan and the secretion of endocan into the bloodstream may be significantly enhanced [23, 51–53]. For example, TNF-α upregulated endocan messenger RNA in human umbilical vein endothelial cells, which could be enhanced by stimulation with E-selectin but not interleukin-4 or interferon-γ [54]. However, the consequent effects of endocan induction may be varied either in vitro or in vivo. It has been indicated that intrinsic endocan may regulate angiogenesis and inflammation in both physiological and pathological contexts, albeit to different extents [22]. Given the complicated clinical condition, the mechanistic impacts of endogenous endocan may be better examined in disease-associated status such as in the presence of CAD.

In the present study, to elucidate the potential pathological role of endocan in clinical setting, EPCs from specific CAD patients were used. It has been demonstrated in our previous study that the surface markers of EPCs are very similar to that of the mature arterial endothelial cells, which may provide a platform to study the potential vascular inflammation in individual subjects [55]. The current findings demonstrated for the first time that compared with the EPCs from non-CAD subjects, EPCs from CAD patients had increased endocan expression together with impaired functional capacity. More importantly, in vitro administration of siRNA specific for endocan significantly reduced endogenous endocan and recovered EPC function with up-regulating angiogenic proteins and down-regulating inflammatory adhesion molecules. Interestingly, our findings are different from the previously mentioned “vasculo-protective” role of endocan in normal physiological condition and also argue for the pathological effects of endocan on angiogenesis [22]. In fact, in the presence of CAD, the EPC function was impaired while the endocan expression was increased. Inhibition on endogenous endocan actually restored angiogenesis capacity and reduced the inflammation in EPCs from patients with stable CAD. Our findings did support the potential pathological role of endocan in clinical CAD.

Given the major role of EPCs in endothelial repair as well as angiogenesis and the significance of EPC dysfunction for clinical CV events [51, 52], the induction of endocan in CAD patients may be not only a prognostic predictor but also a potential contributor to the disease progression. Our findings are compatible to the observations in genetic hypercholesterolemia animals, in which endothelial endocan expression was increased in atheroma [15] and the circulating endocan could be even upregulated early before the development of atherosclerotic plaque [53]. Further experiments with genetically deficient mice may help to confirm the pathological significance of endocan in in vivo atherosclerosis.

### Limitations of the study

The strengths of the current study included the inclusion of a large series of patients with stable CAD, the confirmation study, and the potential pathological role of endocan as shown in the in vitro study. However, there were also several limitations. First, the study included only stable CAD patients and the findings should not be extrapolated to patients in other clinical settings. Second, similarly to most of the previous Biosignature studies, the findings are based on a single measurement on endocan and other biomarkers, that was taken during stable conditions to avoid the fluctuation of plasma endocan during follow-up [29]. Third, there was a relatively low incidence rate of hard CV events in the present hospital-based study compared with previous community-based studies, which might impair the prognostic impacts of endocan on hard events. Future studies using a general larger cohort with longer follow-up period should be conducted to confirm the findings. Fourth, the follow-up duration was limited to 24 months in the present study. However, the current findings were validated in another small but independent patient group with a follow-up period of up to 5-years, suggesting both the shorter and longer prognostic impacts of endocan in clinical CAD. Further studies with even longer follow-up period are still needed. Fifth, in addition to the time factors, study size, there were further differences between the study and validation cohorts. Different from that in study group, not everyone in the validation group received PCI. It might be clinically relevant since the endocan level in the validation cohort seem much lower than in study subjects. More importantly, the measurements of endocan were performed by different assays in this study, that is, Milliplex xMAP kit assays which combined ELISA and flow cytometry from Merck for study cohort, and Sandwich ELISA kits from Abcam for validation cohort. While the former could be applied to a significant amount of samples by simultaneous measurement, the latter may be used to double check for the replication of data in a relatively small amount of study samples. In this study, given the requirement for individual measurements for validation, there was no investigation on the correlation of the two different methods. Future study may be required to determine the correlations between the two different measurements if data summation for analysis are indicated. Taken together, the different levels of mean endocan in these two cohorts may be related to different methods of measurement, sample storage condition (-20 vs -80 degree Celsius) and different baseline characters in which higher incidence of hard CV events (46.1% vs. 19.3%), longer follow-up periods (60 months versus 24 months) and various disease severity could be identified in validated cohort compared to study group. Sixth, Taiwan BioSignature study was about decade ago. The original goal is to find out the novel biomarkers in stable CAD patients. While some well adapted traditional biomarkers such as hs-CRP, NT-proBNP and TNF-alpha were measured together later, unfortunately, cardiac troponins, one of the strongest prognostic factors especially in CAD, was not included. Given that the study was conducted in more than 9 hospitals in different areas around the island, it could be difficult to set up the standard protocol to measure cardiac troponin I or T immediately after blood collection. Future study is required to compare the prognostic value of cardiac troponin and endocan individually for clinical implication. Finally, the EPCs used in the current study were obtained from few patients/subjects. Adequate patients are indicated for further validation on the current findings. Given the potential difference between EPCs and mature endothelial cells, the current findings should be further justified, which may be confirmed with human vascular samples if possible.

## Conclusions

Serum endocan was independently associated with future CV events in stable CAD patients. Inhibition on endogenous endocan improved the dysfunction of EPCs from CAD patients. Endocan might be not only a prognostic indicator for risk stratification but also a potential therapeutic target for vascular protection in stable CAD. Future study is required for further validation on the role of endocan in different ethnic cohorts in different clinical settings.

## Supplementary Information

Below is the link to the electronic supplementary material.

Supplementary file1 (DOCX 133 KB)

## Data Availability

Data was available by request.

## Abbreviations

CAD: Coronary artery disease
CI: Confidence interval
CV: Cardiovascular
ECG: Electrocardiography
eGFR: Estimated glomerular filtration rate
EPC: Endothelial progenitor cell
HDL-c: High-density lipoprotein
HR: Hazard ratio
hsCRP: High-sensitive C-reactive protein
ICAM-1: Intercellular adhesion molecule-1
LDL-c: Low-density lipoprotein
NT-proBNP: N-terminal of the prohormone brain natriuretic peptide
PCI: Percutaneous coronary intervention
ROC: Receiver operating characteristic
SDF: Stromal cell-derived factor
TNF-α: Tumor necrosis factor-α
VCAM-1: Vascular cell adhesion molecule-1
VEGF: Vascular endothelial growth factor
