# Supplementary material for: Novel prognostic impact and cell specific role of endocan in patients with coronary artery disease
Source: Clin Res Cardiol. 2024 May 13;114(8):952–68. doi: 10.1007/s00392-024-02458-7 (PMC12283892; doi:10.1007/s00392-024-02458-7)

**Online Table 1.** Baseline characteristics of study subjects in the validation cohort according to the medium endocan level.

| Characteristics | All  participants  (n=76) | Endocan | | P value |
| --- | --- | --- | --- | --- |
|  |  | <74.8 pg/mL  (n=38) | ≥74.8 pg/mL  (n=38) |  |
| Age (years) | 65.7±10.7 | 63.2±9.8 | 68.1±11.0 | <0.001 |
| Male, no (%) | 53(69.7%) | 30(78.9%) | 23(60.5%) | 0.080 |
| Body mass index (kg/m^2^) | 25.6±3.8 | 25.7±3.9 | 25.6±3.7 | 0.316 |
| Hypertension, no (%) | 51(67.1%) | 19(50.0%) | 32(84.2%) | 0.002 |
| Diabetes mellitus, no (%) | 41(53.9%) | 19(50.0%) | 22(57.9%) | 0.490 |
| Current smoker, no (%) | 37(48.7%) | 15(39.5%) | 22(57.9%) | 0.108 |
| Use of anti-platelet, no (%) | 46(60.5%) | 21(55.3%) | 25(65.8%) | 0.348 |
| Use of ACEi/ARB, no (%) | 28(36.8%) | 10(26.3%) | 8(21.1%) | 0.589 |
| Use of statin, no (%) | 32(42.1%) | 15(39.5%) | 17(44.7%) | 0.642 |
| Fasting glucose (mg/dL) | 120.8±37.0 | 113.7±24.8 | 130.7±46.6 | 0.048 |
| eGFR (mL/min/1.73 m^2^) | 61.1±24.5 | 67.1±22.7 | 57.7±27.0 | 0.016 |
| WBC count (CUMM) | 7182±2152 | 6909±1840 | 7502±2338 | 0.158 |
| Total cholesterol (mg/dL) | 160.9±34.9 | 164.2±34.5 | 158.0±34.2 | 0.203 |
| Triglyceride (mg/dL) | 139.9±80.0 | 137.9±69.2 | 140.3±87.6 | 0.415 |
| LDL-c (mg/dL) | 97.6±31.3 | 102.0±31.4 | 94.7±30.5 | 0.113 |
| HDL-c (mg/dL) | 40.0±12.2 | 42.3±11.4 | 37.2±12.4 | 0.054 |
| Endocan (pg/mL) | 107.2±84.3 | 55.7±10.4 | 158.7±93.7 | <0.001 |
| MACE | 35(46.1%) | 11(28.9%) | 24(63.1%) | 0.003 |

ACEi/ARB: Angiotensin converting enzyme inhibitor/Angiotensin receptor blocker; eGFR: estimated glomerular filtration rate; HDL-c: high-density lipoprotein cholesterol; LDL-c: low-density lipoprotein cholesterol; MACE: major adverse cardiovascular events; WBC: white blood cells.

**Online Figure 1.**

1. Hard cardiovascular events B. Total cardiovascular events


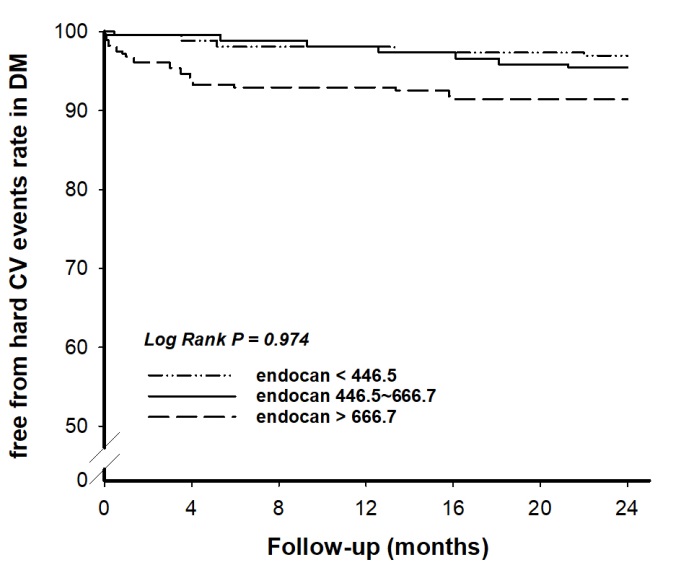

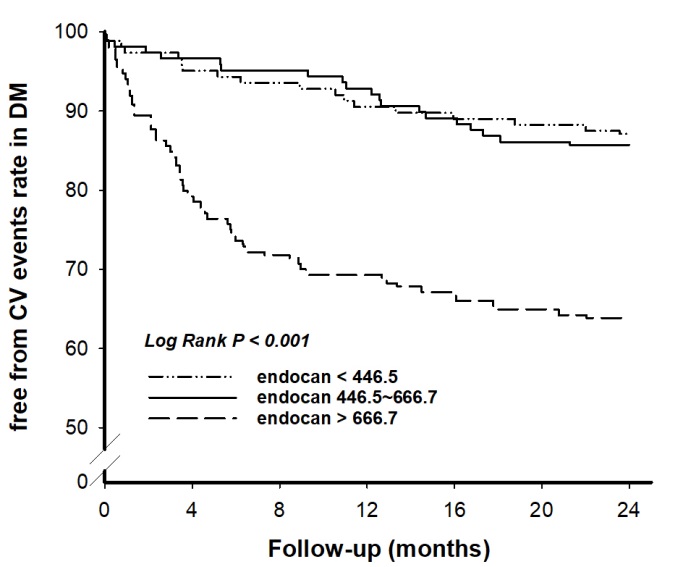

Supplement: Supplementary file 1 — Supplementary file1 (DOCX 133 KB) [file 392_2024_2458_MOESM1_ESM.docx]
